# Critical Thinking and Epistemic Sophistication in Science Education

**DOI:** 10.3390/jintelligence13080093

**Published:** 2025-07-25

**Authors:** Oscar Eugenio Tamayo Alzate

**Affiliations:** Department of Educational Studies, Faculty of Arts and Humanities, University of Caldas, Manizales 170004, Colombia; oscar.tamayo@ucaldas.edu.co

**Keywords:** critical thinking, epistemic cognition, learning, metacognition, metaemotion, argumentation, language, problem solution

## Abstract

One of the central purposes of education at different educational levels is to contribute to the formation of critical thinking in students. There are many theoretical perspectives from which critical thinking is conceptualized, such as those centers on the development of students’ capacities and those based on competences, skills, dispositions and criteria, among others. We consider that in the school context the critical thinking perspective that should come first is the domain-specific one; consequently, we present a conceptual model for the formation of critical thinking in the context of science teaching and learning in the classroom constituted by the integration of four dimensions: languages and argumentation, metacognition, emotions, and problem solving and decision making. Our focus of reflection is epistemic cognition with the processes of epistemic sophistication, metacognitive sophistication, and metaemotional sophistication, determinants of critical thinking in relation to each of the dimensions and the relationships between them. We conclude with the proposal of a conceptual model for the development of critical thinking based on students’ epistemic cognition.

## 1. Introduction

Classroom learning takes place in conceptual, procedural, and value dimensions. Students learn about the fields of knowledge being studied, about human nature, about citizenship, and about coping with adverse situations, among many other kinds of learning. Issues of a conceptual nature are only one of the many types of learning that can be achieved in the classroom. Today, it is not enough to achieve disciplinary, declarative knowledge; students need to acquire the knowledge, skills, and dispositions necessary to live in a dynamic, interconnected, technological, and multi- and cross-cultural world (OCDE 2016; Leu et al. 2018). These challenges that the world offers us every day require the education system, and society in general, to develop in citizens new skills, competencies, and abilities and valuable and fruitful expertise to solve the complex problems that we face today and those to come in a world characterized by uncertainty in many dimensions of human and social development.

Among these new skills are those aimed at developing critical and creative thinking (Bonney and Sternberg 2016; Jenkins 2015; Tamayo 2014, 2021a, 2021b; Tamayo et al. 2014); learning skills for the 21st century, digital and technological literacy, the transfer of skills learned in school, collaborative work (Flanagin and Metzger 2008), learning specialized languages in different fields of knowledge as well as learning about the history and nature of science (Adúriz-Bravo et al. 2023; Duschl 2008; Matthews 2017) are fundamental to understand the diverse dynamics of knowledge construction and to address complex problem solving (Jonassen 2010). Many of these higher-order skills require the development of self-regulation processes that include goal setting, planning, monitoring, and evaluation, among others (Azevedo and Gašević 2019; Gutiérrez de Blume et al. 2022; Schunk and Greene 2017; Tamayo et al. 2023a; Winne 2018; Winne and Azevedo 2022).

The development of these skills has been accompanied by important reforms in the education system, specifically focused on prioritizing actions in terms of making solid progress in achieving deeper understandings of school learning, understandings in which it is fundamental to know how people acquire, understand, justify, change, and use knowledge in formal and informal contexts (Greene et al. 2016). Specifically, in the field of contemporary science education, there is widespread agreement on the need to prioritize the formation of skills in teaching and learning processes instead of continuing with classroom experiences based on the logics of the disciplines taught. Among these efforts, those aimed at training students to think critically, creatively, scientifically, logically, reflectively, and practically, among others, stand out, as well as those experiences aimed at developing higher-order skills such as argumentation, problem solving, and self-regulation. Before proceeding further, it is necessary to point out that these fundamental skills for classroom learning, as the higher-order skills that they are, are both aptitudinal and dispositional in nature.

In the following pages, in Section 2, we will argue in favor of positioning the development of Domain-Specific Critical Thinking (DSCT) as the central object of science education, considering learning and teaching as mediations for the achievement of DSCT. That is, we acknowledge the broad research traditions in the lines of research on learning and teaching science in the classroom; however, we argue for considering these two processes as mediations for accessing the development of critical thinking. In Section 3, we will present the domain-specific critical thinking model with its four constituent dimensions: language and argumentation, problem solving and decision making, metacognition, and metaemotion. In Section 4, we will state some generalities about epistemic cognition in science learning in the classroom. It should be noted that the theoretical perspective assumed throughout the document in reference to the different constructs that developed metacognition, metaemotion, argumentation, and problem solving is of a mixed nature; likewise, we assume a developmental perspective given our interest situated in the classroom. In Section 5, we will present a model of epistemic cognition in which we detail the processes of cognitive, metacognitive, epistemic, and metaemotional sophistication. In this section, we argue for the recognition of epistemic cognitions inherent in each of the constituent dimensions of critical thinking in such a way that the epistemic cognitions used to argue are not the same as those used to solve problems. The developments presented above lead us to the sixth section, by way of conclusion, in which we highlight some of the relationships between epistemic cognition and critical thinking. This section summarizes the model of Critical Thinking in Specific Domains of Knowledge. It is a model that recognizes critical thinking as consisting of four dimensions that act in an integrated manner. Each dimension has at its base the epistemic cognitions inherent to each one of them in such a way that the conjunction of the different processes of cognitive, epistemic, metacognitive, and metaemotional sophistication, in union with the epistemology of the field (e.g., biology) and with the nature of science (e.g., NOS in biology), allows us to access Domain-Specific Critical Thinking (in biology). The qualities of the announced model make it a unique model for understanding domain-specific critical thinking.

## 2. Training in Critical Thinking: The Central Object of Science Education

We believe that one of the central purposes of education at different levels is to contribute to the formation of critical thinking in students. Among the different perspectives on the study of critical thinking, we highlight the one that considers it as a general skill that, once acquired, can be transferred to different domains of knowledge and life in general. Critical thinking has also been considered as synonymous with scientific thinking (Bachelard 1994; Tamayo et al. 2014). A third path in its study has been influenced by liberation theory, with valuable contributions from philosophy, sociology, anthropology, and psychology; this perspective has had important developments in Latin America from the constructs of critical pedagogy (Santos 2003). The fourth way, the one that has had the most decisive impact on critical thinking research, follows pragmatist orientations and characterizes critical thinking in terms of people’s abilities and dispositions (Ennis 1985; Facione 2007; Saiz Sánchez and Rivas 2008, 2012). The four perspectives mentioned above, although they have had important developments according to their possible contexts of application, have not been fruitful in the context of science teaching and learning, aspects that we develop below.

**Figure 1:**
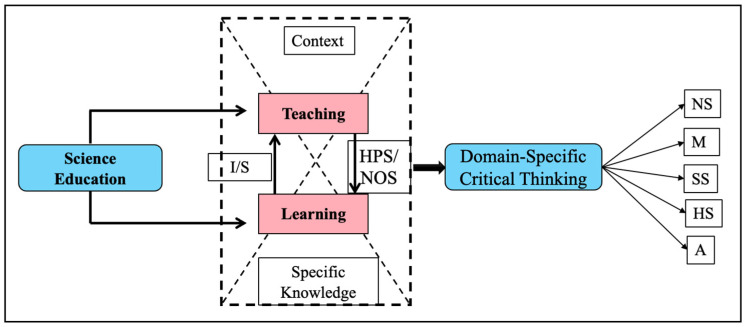
Teaching and learning as mediators in the formation of domain-specific critical thinking (DSCT). The dotted box in the center shows the interaction among context, Individual/Society (I/S), History and Philosophy of Science/Nature of Science (HPS/NOS), and the specific knowledge taught. Domain-Specific Critical Thinking occurs in the different fields of knowledge: Natural Sciences (NS), Mathematics (M), Social Sciences (SS), Human Sciences (HS,) and Arts (A) (Tamayo 2021a).

First, we consider that the central and traditional objects of study in science education, learning and teaching, constitute mediations in the development of domain-specific critical thinking (DSCT). We consider that the central purpose of didactics is not the achievement of student learning; nor is it the achievement of good teaching processes by teachers. We think that the central object of didactics is to train in thinking; in this sense, teaching and learning are constituted as mediations in terms of the development of DSCT. In assuming this perspective, we highlight the existence of substantial differences between critical thinking in the natural and social sciences; in other words, critical thinking is sensitive to the domain of knowledge. Secondly, we value a certain distancing from those theoretical and methodological perspectives that privilege pragmatism and focus mainly on the characterization of abilities and dispositions in the subjects. Thirdly, the theoretical perspective assumed here considers that teaching, learning, and the interactions between them in educational contexts are mediations that contribute to the formation of critical thinking in specific domains of knowledge. For the development of this type of thinking, problem solving, and decision making, the use of languages and argumentation, emotions and motivations, and metacognition is important. The interaction between these four dimensions makes it possible to achieve a holistic understanding of the constitution of critical thinking (see Figure 1).

Some of the central criteria referred to in Figure 1 highlight interactions between the categories of teaching and learning. First, is the necessary interaction between individual and social processes in terms of learning theories and concepts, as well as for the development of other dimensions of human development. Second, is the recognition of the history and epistemology of the fields of study, in our case, the natural sciences and, in particular, the ways of constructing scientific knowledge in the classroom within the framework of the so-called nature of science (Izquierdo-Aymerich and Adúriz-Bravo 2003; Adúriz-Bravo et al. 2023; Matthews 2017). Third, is the recognition of the context as a starting point for warm teaching processes, in which the design of classroom interventions starts from the recognition of socio-scientific problems and considers, in turn, the transfer of learning achieved in the classroom. Finally, is the recognition of the specificity, in terms of teaching and learning, of the different fields of knowledge.

## 3. Critical Thinking in Specific Domains. A Model Under Construction

Various definitions of critical thinking have had an impact in academia (e.g., Bailin 2002; Facione 2007; Ennis 1985; among others). A common feature of these definitions is their generic nature. Given our central interest in science education, we are interested in distancing ourselves from generic perspectives on critical thinking and also in focusing on science learning. Consequently, rather than defining critical thinking, we consider it pertinent to specify some guiding criteria for critical thinking in specific domains of knowledge. That is to say, rather than defining what domain-specific critical thinking is, we consider it more orienting in the science classroom to refer to the criteria and dimensions that make it possible, aspects that we will develop later on.

**Figure 2:**
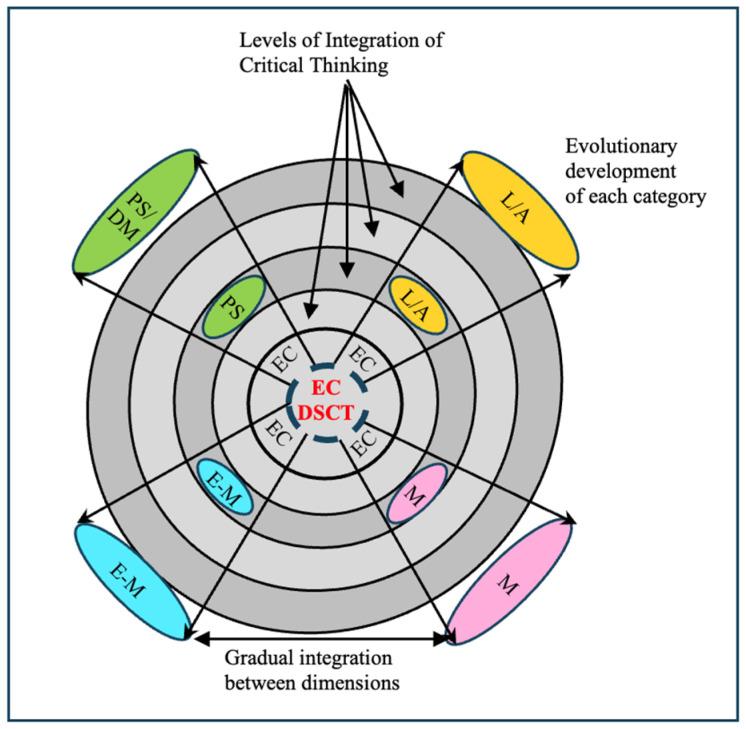
General model for the study of Domain-Specific Critical Thinking (DSCT). The four constituent dimensions of the DSCT model are indicated: Language and Argumentation (L/A), Metacognition (M), Emotions–Motivations (E-M), and Problem Solving (SP)/Decision Making (DM). Epistemic Cognitions (EC) are inherent to each of the dimensions and, when integrated, contribute to the DSCT. The concentric circles illustrate the different levels of education, so that for each level of education, according to the learning objectives, the integration of the four dimensions of critical thinking is expected.

One of the fundamental conditions for the development of critical thinking in specific domains considers the nature of the specific field of knowledge and the new problems that need to be faced today. By way of illustration, in the field of biology teaching today we need to know how to argue, how metacognition and emotions are agentic, how problems are solved, how this field participates in the construction of citizenship, and what are the languages of biology, among many other aspects. According to Tamayo (2021a), thinking critically in biology requires the participation of the dimension centered on the uses of languages and argumentative processes; it also requires taking into account the typology of problems specific to biology and the ways of conceptualizing them and solving them, for example, problems related to evolution in biology. Furthermore, it requires the agency of learning processes, a dimension that relates the student’s learning history and their experience with the nature of biology. The latter aspect is linked to the determining role of emotions–motivations in all human action and, therefore, in those in which people think and act critically in the fields of the natural sciences. The interaction between these four dimensions of critical thinking (see Figure 2) allows for a deeper understanding of student performance and provides possibilities for identifying possible obstacles that may constitute threats within each of the dimensions analyzed or in the interaction between them and which, in turn, allow for guiding educational actions in terms of achieving greater development in terms of students’ critical thinking. At the basis of the development of critical thinking, and of multiple skills and competences, are people’s epistemological beliefs (Muis 2008).

In general terms, there are research results that relate domain-specific epistemological beliefs to student achievement (Hofer 2000; Merk et al. 2018), to conceptual understanding and change (Tamayo and Sanmartì 2007), to argumentation (Iordanou et al. 2016; Ruiz Ortega et al. 2015), and to domain-specific critical thinking (Tamayo et al. 2014; Tamayo 2021a, 2021b), as well as to students’ interest in the topics of study. In this gradual process of learning concepts and theories, solving contextualized problems and advancing in the management of their learning, the child learns to construct knowledge in biology. They learn, for example, the specific characteristics of experimentation in biology, the ways of working scientifically in this field of knowledge, the types of problems investigated, the nature of scientific work in biology classrooms, the diversity of models that explain biology, the ways of communicating using multiple systems of representation, the most fruitful ways of reasoning to adequately respond to the problems studied, and the ways of communicating the results of their biology learning processes.

We consider that the central object of education is to contribute to the formation of critical thinkers. From the field of general and domain-specific sciences, the central object focuses on the reflection on what to do with the knowledge learned in the classroom and how to put it at the service of problem solving in different contexts, aspects that undoubtedly require transforming teaching actions and with them the teacher training processes, as training in critical thinking requires being a critical thinker.

We consider Critical Thinking (CT) as a construct constituted by four dimensions: (a) the uses of languages and argumentation; (b) problem solving and decision making; (c) emotions–motivations; and (d) metacognition. The integration of these four dimensions acquires conceptual and methodological particularities according to the different fields of knowledge: social sciences, human sciences, natural sciences, arts, and mathematics (see Figure 1). Some of the central characteristics of the critical thinking model (Tamayo 2014, 2020, 2021a) proposed are:

- Its constitution from the four dimensions is enunciated.
- The integration of the four dimensions and their evolutionary development according to the different educational levels and fields of knowledge.
- The specificity of critical thinking according to knowledge domains.

We assume in our domain-specific models of critical thinking the gradualness of its development. Critical thinking, like all other socio-cognitive skills, is developmental in nature. Children, young people, and adults can think critically in the different fields of knowledge, if the teaching and learning experiences lived by them at school were planned by teachers with intention and awareness in function of the achievement of this type of thinking. To put it more radically, if teachers are not critical thinkers and the school does not train according to the development of domain-specific critical thinking, students will not be critical thinkers. This model assumes that a good professional is not an enlightened one, knowledgeable in the conceptual field and erudite in his subject; a good professional is one who thinks critically in his field of knowledge, which requires, among other aspects, disciplinary knowledge, self-regulation of his performance, knowledge of the context in which he does his work as a professional, a deep understanding of the situations he intervenes, and his reasonable and autotelic actions.

In the following, we will refer in a general way to the four constitutive dimensions of domain-specific critical thinking (see Bailin 2002; Bailin and Siegel 2003; Tamayo 2020, 2021a, 2021b; Tamayo et al. 2014). This general approach is intended to pave the way in terms of presenting some detailed, guiding ideas in terms of achieving a comprehensive and evolving understanding of critical thinking.

*Languages and argumentation in the formation of critical thinking.* The different languages (Jewitt 2008; Kress 2009; Tamayo et al. 2018), whether understood according to their communicative function, the structuring of thought, or the one that highlights their role in the construction of reality (Berger and Luckmann 1968), are determinant in the teaching and learning processes. Likewise, argumentation in science is today central in science education in terms of its epistemic function (López and Tamayo 2019; Ruiz Ortega et al. 2015; Zohar and Nemet 2002). A teaching that prioritizes the intentional and conscious use of multiple languages and that generates argumentative scenarios brings students closer to the forms of scientific work typical of academic communities. Languages and argumentation are, then, central when guiding teaching actions dedicated to educating critical thinkers.

*Problem solving in the formation of critical thinking.* Critical thinking contributes to appropriate decision making and problem solving. A central purpose of education is to contribute to the formation of people who are good thinkers in the broadest sense of the term: effective problem solvers, reflective, curious, and eager to understand their world; people who have a wide repertoire of formal and informal tools that they use when solving problems (Järvelä et al. 2018; Jonassen 2010; Nickerson 1994; Solano-Guerrero et al. 2024); people who recognize the complexity of a problem, who consider different solution paths, and choose the most successful one based on reasons rather than on sequences or rules.

*Metacognition in the formation of critical thinking.* The learning of metacognitive skills in teaching is considered one of the most dynamic lines of research in recent decades (Thomas 2011; Zohar and Dori 2012). According to Tamayo (2006), metacognition refers to the knowledge, awareness, and control that people have over their actions and thought processes. The evolution in this field of study leads us today to study finer metacognitive processes, for example, calibration and metacognitive judgments, in the learning process (Gutiérrez de Blume and Montoya 2023; Tamayo et al. 2023a), as well as in the development of critical thinking (Bråten and Strømsø 2006; Chevrier et al. 2019; Ford and Yore 2011; Muis et al. 2015, 2018; Sawyer 2014; Taber and Akpan 2016).

*Emotions and the formation of critical thinking.* There is no deep learning in the school context that is not mediated by emotions. In terms of Tamayo et al. (2023b), if we can get our students to involve their emotions in a substantive way in the learning process and, likewise, teachers to do so in terms of their teaching, we would be more likely to achieve deep learning in our students, in other words, learning processes characterized by the appropriate use of languages according to the fields of knowledge, by the achievement of self-regulation processes, and by the transfer of what has been learned in the classroom in order to solve problems of the students’ everyday life, among other aspects (Ramírez Zuluaga and Alzate Tamayo 2011).

The recognition of the emotional dimension in the formative processes in general, and specifically in the constitution of domain-specific critical thinking, becomes more and more determinant every day. All school learning, in addition to recognizing students’ prior knowledge as a starting point for good teaching, takes into account the emotional, motivational, axiological, and procedural aspects of the students. Thus, a field is not learned only by paying attention to its epistemological structure. It is the students, with their interests, motivations, contexts, and previous experiences, who make it possible both to learn concepts and, perhaps more importantly, to perform critically with them.

**Figure 3:**
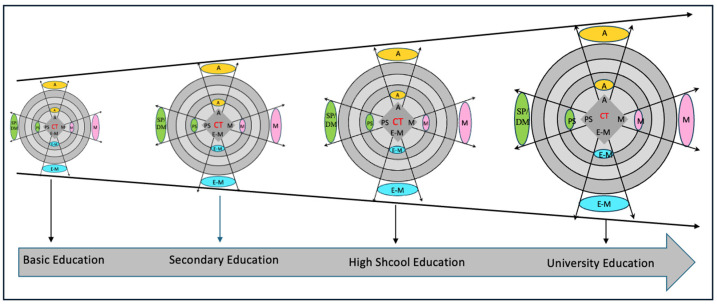
Evolutionary perspective for the development of Domain-Specific Critical Thinking of knowledge. In the proposed model, we draw attention to the integration of the four constituent dimensions of the Critical Thinking model: Languages and Argumentation (L/A), Metacognition (M), Emotions–Motivation (E-M), and Problem Solving (PS) and Decision Making (DM).

As noted above, our model of critical thinking integrates four dimensions. Each of these dimensions is a necessary but not sufficient condition for critical thinking. To illustrate: a teacher or a student can use the languages of chemistry well and argue well in chemistry but not necessarily solve problems well and make good decisions in this field of knowledge. In the proposed model, the four dimensions act as a unit, and it is the synergy between these dimensions that leads to critical thinking. To conclude this section, the evolutionary perspective in the development of domain-specific critical thinking is a task of the general educational system and, specifically, of the specific science education. Figure 3 represents critical thinking as a unit and identifies different levels of complexity corresponding to the different educational levels. Thus, a 10-year-old child can adequately use different languages to refer to cell theory, as well as intervene in argumentative scenarios; he/she can actively participate in the solution of contextualized problems about issues related to this topic in biology; likewise, he/she can gradually advance in the self-regulation of his/her learning processes with the participation of his/her motivations. In doing so, he/she thinks critically in biology. By way of illustration, thinking critically in biology requires:

- Using the modes of language and, in general, the systems of representation with tradition in the field of biology.
- Identifying and solving problems in biology in an appropriate and pertinent manner according to contexts.
- Discussing and arguing in a reasoned manner in relation to theoretical or practical problems in biology.
- Self-regulating learning in this field of knowledge.
- Managing emotions during performance in matters related to biology.

## 4. Generalities on Epistemic Cognition and Classroom Learning

In recent decades, important theoretical and methodological developments have been reported in relation to personal epistemology (Hofer and Pintrich 2002), epistemic cognition (Greene et al. 2008; Kitchener 1983), or epistemic thinking (Kuhn and Weinstock 2012). Personal epistemology is understood as a person’s point of view on the nature of knowledge and knowledge, in particular, that is his or her own. Nature of Science (NOS) refers to beliefs and knowledge about the nature and generation of scientific knowledge (Elby et al. 2016).

One of the objectives of this field of knowledge is to describe and understand the beliefs of people, whether they are experts or novices, about what knowledge is and how it is constructed (Greene et al. 2016), questions that have had answers and contributions from psychology, philosophy, cognitive sciences, specific disciplines (biology, chemistry, etc.), and science education, among others. From our particular interest in the teaching and learning of science, current work on epistemic cognition revolves around three lines of research: (1) personal epistemology, (2) the nature of science, and (3) epistemic cognition and epistemological obstacles. These three lines, which will be presented below, are of particular importance in the educational context and, especially, for the processes of teaching and learning science in the classroom. The first, without losing sight of epistemological reflection in itself, is concerned with understanding the processes that make educational actors think and act in one way or another when they refer to scientific knowledge; the second, is linked to the particularities of school science and school scientific activity (Izquierdo-Aymerich and Adúriz-Bravo 2003; Matthews 2017).

Generally speaking, and by way of illustration, the epistemic cognitions that students have about the Theory of Evolution (TE) largely guide the learning strategies they engage in. Also, if they believe that learning TE is easy, they will devote little effort and time to learning. If students believe that teachers and textbooks already contain all the knowledge about TE, they may simply learn and repeat information. If they consider that there is no connection between learning biology and their emotional and motivational states, they will do little to qualify their learning processes. The following pages develop in detail the aspects mentioned above.

### 4.1. On Epistemology and Knowledge

We do not intend in this section to discuss in detail aspects of an epistemological nature, as they are beyond our interests and capabilities. We content ourselves with presenting some aspects of an epistemological nature that have important implications for the construction of knowledge in the classroom. Epistemology has long been the subject of study and competence of philosophers. Only in the last few decades have psychologists and professionals from other fields of knowledge such as science education begun to investigate individuals’ conceptions of knowledge and knowing as well as their influence on teaching and learning (Abd-El-Khalick et al. 1998; Lederman 2006; Matthews 2017; Tamayo et al. 2010, Adúriz-Bravo et al. 2023). From a philosophical point of view, epistemology has traditionally been used to refer to the nature of knowledge, its limits, and ways of justifying it. Personal epistemology is defined by Hofer and Pintrich (2002) as being concerned with the origin, nature, limits, methods, and justification of human knowledge. According to the authors, in the psychological domain, personal epistemology, or epistemic cognition, refers to what individuals think knowledge is and how they think they and others know or come to know. It refers, then, to how one develops, interprets, evaluates, and justifies knowledge (Greene et al. 2008; Greene et al. 2010; Hofer and Pintrich 1997).

Early reflections on epistemology included views on knowledge and knowing with developmental aspects of affectivity and identity, i.e., they were more holistic reflections. As the field evolved, it focused exclusively on aspects related to beliefs about knowledge and knowing, a trend that continues and in which it is explicitly proposed to restrict personal epistemology to views about knowledge and knowing, in order to align with the philosophical definition of epistemology and thus to achieve greater clarity and enable researchers to communicate effectively (Hofer and Pintrich 1997; Sandoval 2005; Sandoval 2009).

As is typical in these fields of knowledge, this focus of epistemology on the study of beliefs about knowledge and knowing is questioned by various authors. Elby (2009), assuming a more pragmatic perspective in the classroom, proposed that what is important is not so much philosophers’ definitions of epistemology but understanding the complex relationships between, for example, students’ cognitive structure and their relationship to knowledge and its construction, while Chinn et al. (2011) said that restricting epistemology to beliefs about knowledge and knowing is philosophically outdated and advocated broadening its construct to include other dimensions central to knowledge construction in the classroom, such as epistemic goals and epistemic virtues and vices, aspects that we will develop further below.

These questions have led me to consider this broad field that we have called epistemic thinking, epistemic cognition, epistemic beliefs, or personal epistemology (and that some researchers differentiate) as theories in action (Kuhn and Weinstock 2012) in classrooms and that has as its fundamental object to understand how students and ordinary people think about questions of knowledge and its construction and justification (Chinn et al. 2011; Chinn and Rinehart 2016; Greene et al. 2008; Hofer and Pintrich 1997; Hofer and Pintrich 2002; Sandoval 2005). This leads us to focus more on documenting epistemologies in action during learning and teaching activities, given the important influence of teachers’ and students’ epistemological beliefs on their decision making about the actions they take. Let us not lose sight of the fact that these epistemic beliefs, in a non-conscious way, mostly direct the thoughts and actions deployed by the subjects and, in this sense, understanding them in detail from the level of action can provide us with valuable tools in terms of their explicitness and agency.

One of the fundamental themes in educational research, and one that has remained for many decades, is related to epistemology, the study of human knowledge and knowing. The beliefs that individuals have about the nature of knowledge and knowing can be defined as epistemological beliefs (Schraw and Olafson 2003), which are determinant for teaching and learning processes. In terms of teaching, there is a relative consensus on the role of teachers’ beliefs in making decisions about their teaching practices (Jones and Carter 2013) and, more generally, about the theoretical and practical principles or criteria when taking on teaching processes. In the perspective of Hammer et al. (2005), practicing and trainee teachers tend to construct their learning environments according to their epistemological beliefs. Teachers with constructivist epistemological beliefs pay more attention to discussion, interaction, and problem solving by students than teachers with traditional epistemological beliefs (Topcu 2011, 2013).

In terms of learning, studies showed that students’ epistemological beliefs influence their academic performance (Montoya-Londoño et al. 2021; Schommer 1990; Schoenfeld 1983) and their interpretation of information (Schommer 1990). By way of illustration, Hammer (1994) found that physics students believe that to learn this discipline they must follow the guidelines found in textbooks or stated by teachers; in other words, they follow authoritative criteria in their learning. In the field of mathematics, Lampert (1990) and Schoenfeld (1992) concluded that students consider that in this discipline the answer to problems is of a unique nature (Topcu 2013).

The teachers’ epistemic beliefs seem to guide the teaching and learning processes that they develop in their classrooms. Thus, teachers who believe that the scientific method is central to the development of the natural sciences propose teaching science by following the guidelines of the scientific method, which range from the statement of the problem, the selection of variables, the design of experiments, the collection of information, and the analysis and discussion of the information, all in response to a previously stated hypothesis and in relation to which observation is decisive in the process of falsification or verification of the hypothesis. From the place of learning, this teacher generally designs learning strategies based on the development of observational skills in the students, in which the control of one or another variable in the experiment gives indications, for example, of the role of light in the growth of plants.

In summary, epistemology has distinguished different types of knowledge (propositional, evidential, etc.) and justification of knowledge claims (strong or weak arguments that support knowledge, role of evidence, etc.). Now, from research on teachers’ beliefs, i.e., from their personal epistemology, we start from the assumption that individuals have a set of beliefs that affect their decisions and practices in the classroom (Brownlee et al. 2016). From these two places, we argue with Markauskaite and Goodyear (2017) that epistemology is not just a construct that can be used to describe human performance. It makes it possible to take human epistemic agency seriously. It is also a construct that could be enacted purposefully within human performance, that is, in classroom contexts in which the construction of knowledge by teachers and students is privileged. In the following, and due to the importance of epistemic beliefs in educational processes, we turn to develop in some detail reflections focused on teaching and learning processes.

According to Kitchener (1983), the epistemic level of cognitive activities involves personal reflections or thoughts about epistemic assumptions about knowledge and knowing. These cognitive activities that show personal epistemic beliefs about the nature of knowledge and knowing are included in the higher level of cognition that regulates cognitive behaviors such as reasoning, decision making, problem solving, argumentation, and learning in general (Hofer and Pintrich 1997; Kitchener 1983; Muis et al. 2006; Schommer-Aikins 2011; Stathopoulou and Vosniadou 2007; Yang et al. 2013; Yang and Tsai 2010). In other words, the decisions we make that involve higher-order cognitive processes such as those mentioned above are based on epistemic beliefs.

The previous paragraph has important implications in terms of teaching, learning, and the agency that is intended to be built in classroom spaces. Conscious and intentional control over the beliefs we hold as teachers or as learners seems to be the way forward to transform teaching and learning practices (Pintrich 1999; Sinatra 2016; Tamayo 2021a). However, the complexity evidenced to achieve significant transformations in the performance of many teachers, as well as in the learning strategies followed by teachers and students, is largely explained by the experience gained from everyday life, mediated by implicit cognitions and common sense and far from formal educational contexts. It is an experience that ultimately ends up consolidating models of thought and action on teaching and learning that are common sense and over which we have little capacity for agency due to their implicit nature.

It seems clear, then, that reflecting on what counts as knowledge and how to obtain it, i.e., on epistemic cognitions, or so-called personal epistemologies, involves a process of metacognition (Hofer 2004a, 2006, 2016; Kitchener 1983; Kuhn 1999; Tamayo 2006; Tamayo et al. 2023b). Such metacognition underlies the processes of higher-order thinking (struggle with contradictions) and reflection on personal epistemologies advocated by Parkinson and Maggioni (2017). Muis (2007) also supported the idea that metacognitive or self-regulated learning processes are important for changing personal epistemologies. This mechanism of self-regulated learning argues for a focus on explicit reflection, as practicing and trainee teachers, by explicitly reflecting on their personal epistemologies, are testing their knowledge about the nature of knowing and knowledge and about ways of constructing knowledge in school contexts (Adibelli-Sahin and Bailey 2017; Brownlee et al. 2016). This outlines a discussion of the explicit and implicit dimensions of teaching and learning processes, which we will try to elaborate on in another chapter. As noted in previous pages, the evolution of domain-specific critical thinking requires the recognition of aptitudinal as well as dispositional aspects.

### 4.2. The Nature of Science and Epistemic Cognition

In the previous pages, we presented some general reflections on epistemology and knowledge, with the sole purpose of situating a central reflection in this text and, in general, in the of science education, which has to do with the multiple relations between scientific knowledge and its construction, no longer in the field of epistemological discussions but in the contexts of teaching and learning. We referred specifically to research on the Nature of Science (NOS). In the following, we will present in a general way for the field of research in the Nature of Science, a conceptualization that, more than exhaustive, has as its central purpose to study in detail possible relations between the construction of school knowledge, school scientific activity, and epistemic cognition. The study of the relationships between NOS and epistemic cognition seems essential in teaching and learning processes, as well as in those oriented to teacher training.

For Elby et al. (2016), research on NOS emerged mainly from debates about the aims of science education from the philosophy and sociology of science. This led to an ever-constructing consensus on what students should learn NOS, as well as how to incorporate NOS-specific knowledge into the curriculum (NRC 1996). Lederman (1992) defined NOS as the epistemology of science, science as a form of knowledge, or the values and beliefs inherent in scientific knowledge and its development. Lederman considered that the most generally accepted characteristic values and beliefs of NOS are: (a) observation and inference are distinct, as are (b) laws and theories; (c) science is culturally embedded; (d) scientific knowledge is empirical and creative; (e) it is subjective; and (e) it is provisional.

Science education is fundamental to achieving equity and the full development of the potential of individuals and communities. The first quarter of this century presented us, once again, with a global challenge: the pandemic, with its individual, family, social, educational, economic, and general health implications. We are also now facing what is considered by many to be the greatest challenge and problem facing humanity: global warming. Within this framework of challenges and uncertainties, science education is once again a central concern for society, becoming a fundamental element in achieving equity and social justice (Adúriz-Bravo et al. 2023). In coherence with these purposes, teaching and learning processes should be oriented towards the achievement of deeper understandings of science, technology, and their links with society and culture. We consider that this new component proposed for science education, of an openly meta-scientific nature (that is, of critical reflection on science), is the one that would best contribute to achieving greater levels of equity and emancipation in the education system (Adúriz-Bravo et al. 2023).

Traditional approaches to science teaching emphasize a conception of science typical of positivist and neo-positivist currents of thought, with a strong empirical-positivist character (Linn et al. 1991). This perspective shows science as an elitist, ahistorical, static, finished activity with no links to the actors involved in its realization or to the context in which the facts or phenomena took place (Tamayo 2017, 2021a). These approaches focus more on the what of science than on the how and why and, consequently, do not delve into the workings of the scientific enterprise as a profoundly human activity.

Authors such as McComas et al. (2002) state that the understanding of how science works is considered poor among citizens, probably because at all levels of science education the emphasis is placed on the contents of science in its current state, to the total exclusion of considerations of a historical–epistemological nature. This was accompanied by teaching based on textbooks where scientific developments are presented in a ready-made and decontextualized form, thus reinforcing the construction of an image of a cumulative, objective, and monolithic science of scientific knowledge, which is constructed through the scientific method as the sole method of obtaining knowledge.

We believe that these traditional models of science teaching, with an emphasis on declarative knowledge and little reflection on the meta-sciences, only reinforce a deformed image of neo-positivist science, which is precisely the one with which students arrive in the classroom. These teaching models, or scientific stereotypes deployed by teachers, are the product of factors of a very diverse nature, among which genetic, epigenetic, behavioral, and symbolic aspects were proposed (Jablonka and Lamb 2004) and in which the media and social networks are today determining factors in their construction. It is these cultural inheritances largely lived throughout our existence that ultimately end up gradually consolidating our understandings of the world and the actions we deploy in it; they are lived experiences that permanently, both explicitly and implicitly, consolidate, enrich, and crystallize the image of science that citizens have. It is therefore an image of science, culturally constructed and apprehended by each citizen throughout their experience and with the evolutionary tools that we have as a species. The fact that they are cultural models of teaching, learning, science, etc. embodied in the subjects undoubtedly means that the processes that lead to their change are highly complex, hence the high stability of a cumulative, objective, and monolithic image of science and scientific knowledge.

These were linked to another controversy about how to teach concepts related to NOS, whether they are best learned through explicit instruction (Abd-El-Khalick and Lederman 2000) or by immersing students in scientific enquiry (Sandoval 2005), with concomitant disagreements about the most appropriate conceptual frameworks and outcome measures. Sandoval calls for the study of ‘practical epistemologies’ (epistemic cognition in situ) to advance our understanding of how to promote NOS in schools.

These models of an inductive nature and constructed via experiential processes have been and are the rule of phylogenetic and ontogenetic development. Focusing only on citizens, teachers, students, and specialists at the highest level had (or has) misconceptions in their fields of study. The change in these conceptions into scientific models, through intentional and conscious processes and in function of the achievement of deep learning, requires the participation of high-order socio-cognitive processes, which are, likewise, an evolutionary product. As we will argue throughout this text, the processes of sophistication in three dimensions, epistemic, metacognitive, and emotional, seem to be fundamental in the change in traditional models of teaching and understanding science and scientific schoolwork. In particular, and in relation to epistemic sophistication, epistemic cognitions would hypothetically be responsible for teachers’ and students’ beliefs about what scientific knowledge is and how it is constructed and would also point to a new place in terms of where to gather efforts to achieve the desired change in traditional teaching models. They would also constitute a possible answer to the question of the difficulty of change, for example, about the image of science, as pointed out by Elby et al. (2016) when they stated that both students and teachers consider scientific knowledge to be objective and absolute and difficult to change in them.

Since our work (Adúriz-Bravo et al. 2023), the research that followed on the (meta)conceptions of science in both students and teachers led to the construction of the concept of the nature of science, which refers to a very fruitful line of science education research in recent decades (Lederman 2006; Matthews 2017). This line of research investigates the images of science and the scientist in different audiences and proposes devices and strategies to change these elitist images for more democratic ones. In addition, NOS also refers to an emerging component of the science curriculum; in the latter sense, NOS is a set of contents from the philosophy, history, and sociology of science (McComas et al. 2002) with value for science education, appropriately selected and transposed, to be taught at different educational levels (Adúriz-Bravo et al. 2006).

The incorporation of NOS into science education in schools has been widely accepted by organizations such as the NSTA (National Science Teachers Association 1982) and the AAAS (American Association for the Advancement of Science, 1990), which stated that “A proper understanding of NOS allows an understanding of the empirical and tentative nature of scientific knowledge and an appropriation of the central role of theory and research in science” (Abd-El-Khalick and Lederman 2000, p. 2).

NOS research is also combined with contributions from psychology and cognitive sciences to construct educationally valuable answers to questions such as: what is science, what relationships can be established between science and its teaching and learning, how do scientists operate as a social group, and how does society influence and is society influenced by scientific endeavors? It is precisely in relation to the cognitive sciences that we propose developments referring to the processes and dimensions of sophistication: epistemic, metacognitive, and metaemotional. Specifically, at this point in the text, it is of particular interest to advance some reflections on the epistemic cognition of teachers, students, and, in general, citizens. To advance in this purpose, we will present some central ideas around the concept of an epistemological obstacle, a concept with an important theoretical tradition and which could provide us with an anchorage point for the subsequent discussions that we will propose.

### 4.3. Epistemic Cognition and Epistemological Obstacles

In the processes of teaching and learning school science, three processes are fundamental: first, an adequate transposition of theories, concepts, and forms of scientific work, processes that refer to the NOS, already outlined in previous pages and for which teachers are mainly responsible; second, the identification of obstacles of different nature, among them epistemological ones, and against which both students and teachers participate in their identification; third, the epistemic sophistication achieved by teachers in their teaching action and its correlation with the epistemic sophistication achieved by students in their learning action. The integration of these three processes, taking the concept of an obstacle as the axis, will be the purpose of the following paragraphs.

As presented in previous pages, epistemological, cognitive–linguistic, emotional–motivational, and metacognitive obstacles are integrated in terms of the achievement of deep learning in students (Tamayo 2009, 2017). Of this set of obstacles, the epistemological ones are the ones that have had more tradition in their study and had as a starting point the notion and the processes of construction of scientific knowledge, from different theoretical perspectives. For the empiricists at the end of the 19th century, knowledge is constructed from the senses, alluding then to sensual empiricist knowledge; in this perspective, knowledge is external to the subject and both scientific and everyday knowledge are produced from observation. This understanding of knowledge and its construction took shape in teaching models that privileged observation. From the 20th century onwards, and due to scientific and technological advances, as well as the emergence of new currents of thought, the so-called inherited conception entered a crisis (Adúriz-Bravo et al. 2023).

Bachelard believes that epistemological obstacles must be taken into account in the process of knowing. In this respect, he states that, when investigating the psychological conditions of the progress of science, one soon comes to the conviction that the problem of scientific knowledge must be posed in terms of obstacles (Bachelard 1994). For this author, obstacles are not a lack of knowledge; on the contrary, they are knowledge or knowledges of great utility for subjects, which serve to face and solve certain problems but which, in other contexts, specifically when they come into contact with scientific knowledge, are the first answers that appear and may be inappropriate, imprecise, false, or inadequate in these new contexts. Nor is it in our interest to understand the obstacle as something that does not allow us to move forward, as something that obstructs the achievement of a given purpose and against which there is little to do. As in any obstacle race, it is necessary to recognize them, observe them, rationalize them, understand them, and feel them; only in this way can the obstacles be intentionally and consciously transformed into mediations to advance in the task. Common, concrete, real, natural, and immediate experience becomes an obstacle when there is no action that relates it in any way to the scientific experience of the science classroom.

In general, the literature built around the concept of obstacle has been strongly influenced by Bachelard’s (1994) “The Formation of the Scientific Spirit” (see Astolfi 1999; Cuéllar Alzate 2022; García Cruz 1998; Malisani 1999; Mora 1999; Tamayo 2009). Between the 1980s and 1990s, research interest in science education expanded from the investigation of mere misconceptions to the characterization of genuine epistemological obstacles (Bachelard 1994; Camilloni 2001; Astolfi 1999); the populations under study were also extended from students to science teachers (Porlán Ariza et al. 1997). The new findings showed that science teachers also held naïve ideas that responded to common-sense knowledge. Such ideas, which can strongly guide teachers’ professional performance in the classroom, referred not only to the phenomena of the natural world but also to teaching and learning.

Among the findings of this research, and on which there is general consensus, we can mention the stability of knowledge resulting from basic and everyday explanations in citizens in general; the lack of knowledge of the language of science and its assimilation with everyday language; the presence of cultural and emotional charge; the explanation of scientific phenomena from analogies and metaphors based on familiar ideas; and the spatial-temporal contiguity in the explanation of scientific phenomena; among many others.

Bachelard proposed to approach knowledge in terms of obstacles, in the face of which it is necessary to guide actions to overcome them. The Bachelardian idea of vigilance of thought gains strength when students’ previous ideas about a given concept are identified; through this vigilance, students can self-regulate the expression of their previous knowledge and, with it, of the obstacles to learning. In the terms of Fabre (2005), only with a reflective distancing of the obstacle does it become identifiable as such (Orrego et al. 2016). It is therefore necessary to create teaching situations that encourage students to become aware of the obstacles involved in their own thinking.

There are general epistemological obstacles, transversal to different fields of knowledge, and domain-specific ones, inherent to a given field of knowledge. Moreover, it seems clear today that domain-specific epistemological obstacles change according to the subjects studied; in other words, the epistemic cognitions hypothetically responsible for epistemological obstacles are situated and the product of individuals’ experiences. About the former, Astolfi (1999) illustrated the mental functioning of the obstacles based on research carried out on the analysis of the transformations of matter, both physical–chemical and biological. In these studies, alternative conceptions or similar ways of thinking were found, even when the students were confronted with different scientific fields. As for the latter, domain specificity, the obstacles to the construction of biological knowledge are different when learning respiration, immunity, genetics, etc. (Orrego et al. 2016; Orrego Cardozo et al. 2013).

It is clear then that when speaking of obstacles in the construction of scientific knowledge, some are obstacles to learning chemical knowledge and others, biological; some are obstacles to inheritance and others are obstacles related to mental models of respiration. Now, for Astolfi, different conceptions in different fields of knowledge can be points of emergence of the same obstacle of a general nature, which makes it necessary to identify and recognize the obstacles that underlie them. Below, we describe some typologies of obstacles to learning based on developments presented elsewhere (see Orrego et al. 2016).

## 5. Epistemic Cognition and Processes of Epistemic, Cognitive, Metacognitive, and Emotional Sophistication

We argued so far that school learning and teaching are constituted as mediations or knowledge artifacts (Knuuttila 2021; López and Tamayo 2019) in terms of the development of Domain-Specific Critical Thinking (DSCT). We also highlighted the determinant role of teachers’ and students’ epistemic cognition on the construction of school knowledge. Based on these two groups of reflections, we will now focus on the processes of epistemic, cognitive, metacognitive, and metaemotional sophistication, which are based on the epistemic cognitions of teachers when teaching and of students when learning.

In turn, this section constitutes the preamble that leads us to defend the thesis in which we argue that it is the epistemic cognitions of the subjects that are responsible for their performance as critical thinkers in specific domains of knowledge. Likewise, we argue that the path to the achievement of domain-specific critical thinking requires the processes of sophistication enunciated in the title of this section and developed below. Illustrated with two examples inherent to the argumentation and problem-solving dimensions of the critical thinking model presented above, we learn to argue only to the extent that we become more sophisticated in our epistemic cognitions about argumentation and we learn to solve problems only to the extent that we become more sophisticated in our epistemic cognitions about problem solving. In brief, we learn to think critically only to the extent that we sophisticate our epistemic cognitions about what it is and how to think critically in specific domains of knowledge. By way of example, only to the extent that we intentionally and consciously take hold of our explanatory models about the origin of life, be they common sense, mythological, or religious, and that we accompany this agency with others of a metacognitive and emotional nature will we be able to advance in our abilities to think critically in biology.

**Figure 4:**
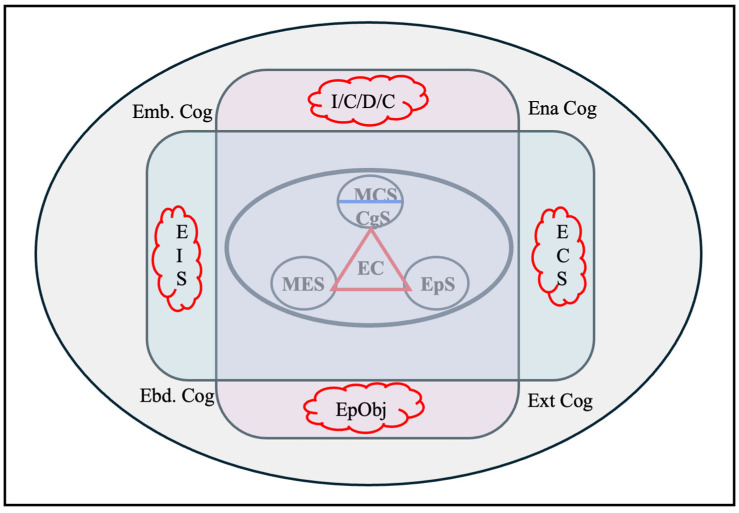
Model of epistemic cognition (epistemic beliefs/personal epistemology). The interactions between Metacognitive Sophistication (MCS) and Cognitive Sophistication (CgS) and between Epistemological Sophistication (EpS) and Metaemotional Sophistication (MES), which are determined by the subjects’ Epistemic Cognitions (EC), are depicted in the center of the figure. The interactions between these processes, enclosed in a black ellipse, constitute the bridge between two processes in the classroom. The first, in the horizontal, relates the Emotional Interaction Space (EIS) and the Emotionally Competent Stimuli (ECS) generated in the classroom by virtue of the interactions between teachers and students. The second, in the vertical, highlights the interactions among Intention, Conscientiousness, Disposition, and Commitment (I/C/D/C) with the epistemic goals during the learning process. The outer gray ellipse frames the theoretical perspective taken from Embodied Cognition, with its four characteristic components (Embodied (EmbCog), Embedded (EbdCog), Enactive (EnaCog), and Extended (ExtCog)). Emotional Interaction Spaces (EISs), Emotionally Competent Stimuli (ECS).

In science classrooms, the joint construction of Emotional Interaction Spaces (EIS) (Tamayo et al. 2023b) has as its telos the construction of concepts, theories, and explanatory models (see Figure 4). Undoubtedly, there are many other purposes that are pursued in science education, but those specifically related to science learning are the ones that concern us at this time These EISs are by nature ideal chronotopes for the expression of points of view on the issues studied in the classroom, expressions of thoughts, beliefs, experiences, intentions, and emotions, related, explicitly or implicitly, to the construction of knowledge. They are, then, epistemic thoughts (Barzilai and Zohar 2016), which are consistent with an important set of experiences and conceptualizations oriented towards the formation of thought constructed in recent decades (Dewey 2022; Gardner 2009; Kuhn 2009; Perkins 2017).

Focusing on epistemic thinking invites us to focus our attention not on thinking as a whole but on those thoughts involved in the construction of scientific knowledge. These are thoughts that include conceptual (epistemic), cognitive, metacognitive, and emotional dimensions; in this sense, understanding how students think when learning science requires us as teachers to make sense of the complex interactions between the four dimensions outlined above. In agreement with Barzilai and Zohar (2016), we see the educational goal of developing epistemic thinking as an extension of the tradition of research oriented towards education for thinking.

Assuming that epistemic cognition is situated is consistent with the theoretical perspective presented below (see Section 5) in which we defend the idea that epistemic beliefs underlie the processes of sophistication in the four constituent dimensions of the critical thinking model presented here. In other words, students’ epistemological beliefs are situated and appear to be determinant in students’ argumentative performances and the function they assign to language in the science classroom, in the metacognitive processes they engage in, in the ways they solve problems and make decisions, and, furthermore, they are determinant in the achievement of emotional sophistication. What we propose here undoubtedly generates important challenges for researchers and teachers in terms of understanding the sophistication processes of each of the four dimensions mentioned and, likewise, of the sophistication of critical thinking as a unit.

The conceptual dimension of epistemic cognition refers specifically to those thoughts, beliefs, or experiences referring to phenomena of a scientific nature. For example, explanations, understandings, or experiences related to breathing, global warming, or biological evolution illustrate this dimension. The cognitive dimension of epistemic cognition refers to those thought processes based on skills such as analysis, explanation, reasoning, and observation; it is thinking based on the epistemic status and properties of specific information, knowledge claims, and their sources, as well as engaging in epistemic strategies and processes to reason about specific information, knowledge claims, and sources. These are, then, first-order epistemic cognitions that denote the cognitive or strategic level of epistemic thinking; in our examples, what cognitive processes do students employ to refer to breathing or global warming? The metacognitive dimension of epistemic thinking refers to thought processes in which we think about our own thought processes. It is, then, meta-level thinking; its status and properties are related to the achievement of epistemic ends, such as truth, error avoidance, justification, and understanding (Barzilai and Zohar 2016). In our example, when we solve a problem about biological evolution, how do we monitor the process of solving the problem? Finally, the emotional dimension of epistemic thinking refers to all those processes responsible for the agency and modulation of emotions throughout the process of learning about issues related to, in our example, evolution or global warming, guided by questions such as: how do I manage to transform epistemic emotions of negative valence when learning about evolution into emotions with positive valence?

It seems clear that epistemic cognition is thinking of a metacognitive nature, a meta-level thinking associated with metacognitive development, or specifically linked to metacognitive knowledge (Kuhn 1999; Moshman and Tarricone 2016) or to sub-dimensions of metacognitive regulation such as evaluation (Barzilai and Weinstock 2015; Hofer 2004b). In the epistemic thinking–metacognition relationship, there are many aspects linked to knowledge. Among them, we can highlight achieving the transition from dualistic to relativistic thinking; managing to think about thinking; moving from being knowledgeable about information to thinking critically about this information; carefully reviewing the sources of knowledge; recognizing that the construction of knowledge requires intellectual energy; and making reference to the nature of knowledge, truth, and justification. This leads us to affirm the closeness between epistemic thinking and metacognition. As meta-level thinking, epistemic thinking is a meta-thinking in which metacognitive actions act on the cognitive processes necessary for the construction of scientific knowledge in the specific domain. In another way, the achievement of deep learning about the theory of evolution, breathing, or global warming is achieved to the extent that metacognitive agency is achieved in relation to the uses of specialized languages, previous ideas, or mental models of ontologies different from those taught in the classroom that are consciously inhibited, suspended, or suppressed (Pozo 2017) and the explanations or arguments given are evaluated, as well as the solution given to the problems.

It also seems clear that epistemic thinking is cognitive thinking. Thinking about knowledge construction involves employing the first-level cognitive structures and processes referred to above. The value of epistemic thinking derives not only from meta-level knowledge but also from how people deal with epistemic challenges in particular academic and everyday contexts. That is, it seems necessary to know the ways in which specific thinking is activated in specific contexts, situations, and domains of knowledge (Chinn et al. 2014; Hammer and Elby 2002). This has led to more attention being paid to epistemic practices, to what teachers and students do and not just to their knowledge and beliefs. This epistemic thinking in action has shed light on various cognitive processes that give rise to epistemic judgements and to the proposition that epistemic thinking operates at both cognitive and metacognitive levels (Barzilai and Zohar 2014, 2016; Richter and Schmid 2010).

Complementary to what was mentioned in the previous paragraphs, the meta-level epistemic understandings that students construct are formed through the repetition of cognitive-level epistemic processes and strategies. We therefore agree with Barzilai and Zohar (2016) when they stated that there is a need to know in detail the cognitive aspects of epistemic thinking and that the distinction between the cognitive and metacognitive levels of epistemic thinking seems necessary to explain the ability to reflect on epistemic processes and strategies, judge their reliability, and thus improve epistemic agency. That epistemic thinking is both cognitive and metacognitive tells us, on the one hand, about the iteracity, complementarity, and transition between cognitive and metacognitive processes and, on the other hand, about the importance of recognizing their scope either in terms of situating reflections in the place of the cognitive, in that of the metacognitive, or in that of agency in relation to the construction of knowledge: epistemic sophistication.

As we can derive from the previous paragraph, epistemic thinking, cognition, and metacognition are imbricated constructs: epistemic thinking implies both cognition and metacognition; however, we already pointed out elsewhere that not all metacognition is epistemic, just as not all cognition is epistemic, just as not all thinking is epistemic. In the study of the relationships among epistemic thinking, cognition, and metacognition, different models were proposed, some on the side of identifying the constituent dimensions of epistemic thinking (Hofer and Pintrich 1997), another focused on understanding it as a complex of cognitions such as true beliefs, justified beliefs, understanding, and knowledge and their representations related to the achievement of epistemic purposes (Chinn et al. 2011). In this case, epistemic thinking is more oriented by the ends to be achieved than by its form or structure, which allows exploring the multiple ways in which people think about epistemic issues, as well as distinguishing between epistemic and non-epistemic cognition and metacognition (Barzilai and Zohar 2016).

With what has been said so far, it seems clear that metacognition is a skill that can be at the service of a very broad spectrum of human and social action and thought and that, when this skill is put to the service of knowledge construction, we delimit its focus of action in what we have called epistemic metacognition. These Epistemic Metacognitive Skills (EMS/EMS) include skills related to metacognitive regulation such as planning, monitoring, and evaluation related to the processes and strategies used in the construction of knowledge; in this sense, they are skills oriented towards the achievement of epistemic objectives. By extension of Flavell’s (1979) seminal proposals, the HMEs could include, in addition to those already referred to in the context of epistemic metacognitive regulation, those alluding to epistemic metacognitive awareness and epistemic metacognitive knowledge type skills; they can also serve both self-regulation and social regulation (Efklides 2008) and be used to regulate other people’s epistemic thinking or to co-regulate collective activity (Grau and Whitebread 2012; Solano-Guerrero et al. 2024).

As for Epistemic Metacognitive Knowledge (EMK), after having pointed out that not all metacognitive processes are epistemic in nature, it should be noted that this refers to the beliefs, knowledge, or theories that people have about the construction of knowledge; that is, knowledge about the individual as a knower, about other people as knowers, and in general about human knowledge. It is therefore meta-level metacognitive knowledge. GCE also considers metacognitive knowledge of epistemic strategies and tasks which includes knowledge about the strategies of knowledge construction and justification, knowledge about the epistemic nature of tasks, and knowledge about epistemic metacognitive experiences, the latter being considered as experiences that are evoked during the processes of knowledge construction and justification and that are related to the nature of knowledge and knowing (Efklides 2008, 2009). Precisely, it refers to knowledge about how to perform a knowledge-conducive task, which implies knowing when, why, and how to use one or another strategy and which of these strategies are more reliable in terms of learning. As might be expected, as a function of knowledge construction, CME imbricates different facets: individual, people, tasks, and strategies (Barzilai and Zohar 2016; Bromme et al. 2010; Muis 2007).

An important finding in the last decade that relates epistemic beliefs to EMK describes that epistemic beliefs predict epistemic emotions and that these, in turn, predict cognitive and metacognitive learning strategies (Muis et al. 2016).

To summarize, the metacognitive agency required for the construction of knowledge in the classroom demands the following aspects from teachers, among others:

- Acting intentionally based on students’ prior knowledge and ways of thinking to achieve better understanding of what is being studied.
- Encouraging the explicit engagement of metacognition. A good metacognitive teaching process recognizes the importance of guiding actions aimed at helping students build and refine their epistemic metacognition, that is, providing them with opportunities to multimodally represent the concepts studied and to solve problems, reflect, plan, monitor, and evaluate their knowledge and metacognitive skills. In other words, ensure that the Emotional Interaction Space (EIS) becomes the support, the scaffolding, that constitutes metacognitive sophistication.
- Plan and carry out their teaching in terms of the achievement of deep learning by students. This requires recognizing students’ initial explanatory models, identifying potential barriers to learning, planning metacognitive teaching focused on diverse activity systems, and ensuring that the expenditure of intellectual energy is primarily undertaken by students, aspects contributing to the agency of metacognitive processes during learning.
- As we already pointed out in other parts of the text, the actions deployed by teachers in relation to metacognitive reflection and action must consider both general and specific aspects. In this sense, teachers should pay attention to domain-specific epistemic metacognition in the facets outlined in previous paragraphs as well as to general metacognitions. Achieving this recognizes an important quality of deep learning: in this case, the two-way transfer between general and domain-specific metacognitive sophistication that is so much in demand today (Chinn and Buckland 2012; Chinn and Rinehart 2016; Morehead et al. 2019; Krieger et al. 2022; Solano-Guerrero et al. 2024).
- To recognize that both epistemic metacognitions centered on the subject, as well as all those others referred to different systems of activity (the dyad, the focus group, the classroom, the institution, the culture, among others), interact in function of the achievement of processes of epistemic metacognitive agency.
- Given the historical tendency to study subject-centered metacognition, we advocate today for the achievement of a dynamic balance among these different activity systems, a balance in which social and collaborative learning, with its characteristic complex interactions between cognitive, social, emotional, motivational, and contextual categories (Järvelä et al. 2018; O’Donnell and Hmelo-Silver 2013), contributes to the achievement of more holistic understandings of deep learning.

## 6. Critical Thinking and Epistemic Cognitions in the Classroom: Interwaving of Cognitive Sciences, Epistemology, and Science Education

In the classroom, the action of teachers integrates, in terms of knowledge construction, cognitive sciences, epistemology, and science education. From our explicit interest—didactic action oriented towards specific training in fields of knowledge, mediated by good teaching and the achievement of deep learning—it incorporates contributions from the other two fields mentioned above. In this section, we propose to construct some conceptual tools to guide the performance of teachers based on the fundamental epistemological criteria of school science and school scientific activity. We will also draw on some of the most fruitful developments in the new cognitive sciences and, in particular, in the science of learning, in such a way that these contributions can be used to guide teaching processes.

In a previous study of just over two decades ago (Adúriz-Bravo et al. 2001) carried out with teachers in training for children, we discussed teachers’ knowledge of science teaching and learning. Already in this study we confirmed, based on the results obtained and in line with other authors (Bråten et al. 2011; Chinn et al. 2011; Muis 2004), that teachers’ ideas about science teaching and learning stem from their beliefs about what knowledge is and how it is constructed. That is, an understanding that scientific knowledge is constructed through the application of the scientific method guides the trainee teachers’ teaching actions dedicated to the achievement of scientific skills in their students and, likewise, they propose learning tasks in coherence with their understanding of the construction of knowledge with the application of the scientific method. Similar understandings were observed with other understandings of knowledge construction, for example, through observation, in which teaching and learning processes were derived by giving special importance to the development of observational skills in students.

**Figure 5:**
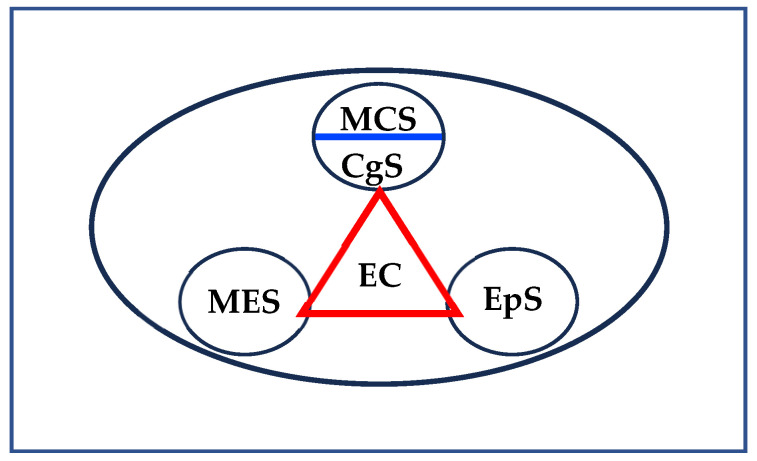
We represent epistemic cognition (EC) as responsible for the processes of metacognitive (MCS), cognitive (CgS), metaemotional (MES), and epistemic sophistication (EpS). In red we highlight the interactions between the three sophistication processes. The blue line highlights the close relationship between cognitive and metacognitive sophistication.

To advance in these reflections, we return to Figure 4 and focus our attention on the center of the image where we highlight the processes of sophistication of interest to us, metacognitive (SMC), metaemotional (SME), and epistemic (SEp), and we situate the epistemic cognition (EC) of the subjects (see Figure 5) as responsible for these processes of sophistication.

Initial work on epistemic sophistication was oriented towards the recognition of students’ beliefs about the structure of knowledge, its source and justification, and its virtues and vices of knowledge are true or tentative, whether beliefs about whether learning is easy or difficult (Elby and Hammer 2001; Hofer and Bendixen 2012). We agree with Elby et al. (2016) that considering epistemic sophistication disjointed from the context in which it is expressed seems simplistic. This perspective, from our specific interest in learning science and understanding the nature of science, becomes important insofar as problem solving, the uses of languages and argumentation in the classroom, and emotions are determinant, from our model of critical thinking, in terms of the formation of critical thinkers in specific domains of knowledge.

It is the students’ epistemic cognitions that determine, in the first instance, their beliefs about how they learn, what to pay more attention to when they are studying, whether or not they recognize the difficulty of the concepts studied and, in general, how they self-regulate their learning. For their part, as we mentioned in previous paragraphs, it is the teachers’ epistemic beliefs that determine, in the first instance, their understandings about teaching, assessment, how to use the models, experience, emotions, interests, etc. that students already bring with them in order to carry out more fruitful teaching processes.

This epistemic cognition that each of us carries with us is the product of both phylogenetic and ontogenetic evolution. It is clear that as a species we inherit from our ancestors ways of operating cognitively, ways of relating to our environment, and ways of going through the world that make the subsistence of the individual and the species more likely. We also inherit behaviors, myths, ideologies, customs, narratives, and discourses (Jablonka and Lamb 2004; Melìch 2019), which, to a large extent, guide the ways of thinking and acting of both individuals and communities.

**Figure 6:**
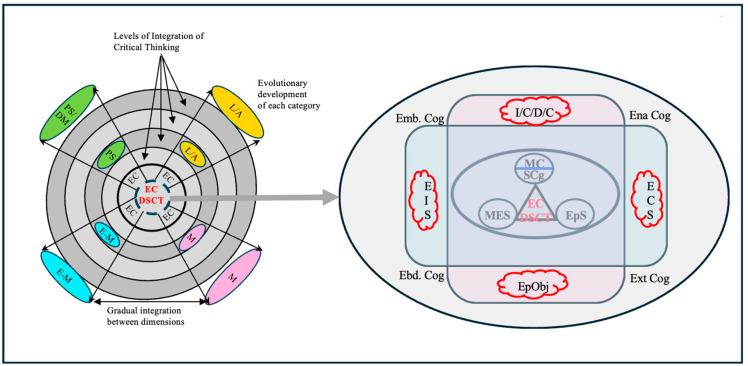
The Critical Thinking (CT) model described above with its four dimensions is depicted on the left: Languages and Argumentation (L/A), Metacognition (M), Emotions–Motivation (E-M), and Problem Solving (PS) and Decision Making (DM). These four dimensions, and the interactions among them, are determined by the Epistemic Cognition (EC) of the subjects, represented on the right; at the base of the processes of Metaemotional (SME), Metacognitive (SMC), Cognitive (SCg), and Epistemic (SEp) Sophistication are the Epistemic Cognitions of the subjects. By virtue of this, thinking and acting critically in specific domains is determined by people’s epistemic cognitions. (Figure 4 presents, in more detail, the description of the other aspects referred to in this figure.)

What is stated in the previous paragraph leads us to foreground the personal epistemology, or the epistemic beliefs of prospective and practicing teachers, as the determining dimension of their models of teaching, learning, and assessment that they employ in the classroom. Likewise, this personal epistemology guides teachers’ understanding of science and its construction in the classroom, that is, the nature of science. Our central purpose in this section is to advance reflections of metacognitive nature oriented by both aptitudinal and decisional aspects the achievement of intertwined processes between the three theoretical categories already mentioned, cognitive sciences, epistemology, and science education, reflections that will qualify the direction exercised by personal epistemology on the understanding of NOS, learning, and teaching. Consequently, the intertwined agency between science, its learning, and its teaching is achieved. The intertwined action between epistemic and metacognitive sophistications is the guarantor of conscious and intentional learning processes and, in turn, of balanced teaching processes in which diverse understandings of NOS interact with other understandings of learning and teaching. This process of sophistication that relates cognitive, epistemological, and didactic aspects is, then, a second-order process that can explain issues related to science teaching and learning in the classroom and, in turn, fulfils epistemic functions insofar as it succeeds in transforming personal epistemology (see Figure 6).

In the previous figure, it is clear that the gradual evolution of each of the constituent dimensions of critical thinking, Languages and Argumentation (L/A), Metacognition (M), Emotions–Motivation (E-M), and Problem Solving (PS) and Decision Making (DM), is necessarily based on the Epistemic Cognitions of students and teachers. In other words, and by way of illustration, the Epistemic Cognitions on language and argumentation, as well as on the other three dimensions presented in the figure, are based on specific Epistemic Cognitions for each dimension (Figure 6). On the right side of the figure, the reasoning is equivalent: the development of Critical Thinking in specific domains of knowledge is supported by Epistemic Cognitions and the processes of Metaemotional (SME), Metacognitive (SMC), Cognitive (SCg), and Epistemic (SEp) Sophistication.

In the model of Critical Thinking (CT) that we propose, epistemic cognition is determinant in the evolutionary development of each of the dimensions of critical thinking, as well as the interactions among them, in terms of the achievement of critical thinking in specific domains of Knowledge (see Figure 6). Likewise, epistemic cognitions are determinant for the achievement of the processes of epistemic and metacognitive as well as metaemotional sophistication required for the evolution of critical thinking in the classroom. The satellite aspects represented in Figure 6 contextualize the reflection on critical thinking in specific domains from Embodied Cognition, a theoretical perspective assumed for teaching and learning in classrooms.

Throughout the text we defend the thesis that the central purpose of didactics is the formation of Domain-Specific Critical Thinking (DSCT). To this end, we start by arguing in favor of considering that learning and teaching in the classroom constitute mediations for training in critical thinking. In line with what was already pointed out, we move forward in the presentation of a model of critical thinking, which we consider to be made up of four dimensions: Languages and Argumentation (L/A), Metacognition (M), Emotions–Motivation (E-M), and Problem Solving (PS) and Decision Making (DM). We also consider that critical thinking should be understood as an integrated construct, as a holistic construct, in which the integration of the four dimensions with different levels of complexity can be studied evolutionarily.

All this is in order to specify that at the basis of critical thinking and each of its dimensions are the Epistemological Cognitions of students and teachers. It should be noted that in the last decade there has been a growing interest in the research community in describing and understanding the role of epistemic cognitions in some of the agentic dimensions discussed (Chevrier et al. 2019; Iordanou et al. 2016; Muis et al. 2015, 2018; García-Carmona 2023) and not so much in understanding the multidimensional Critical Thinking defended throughout this text. Advancing in the understanding of the processes of cognitive, metacognitive, metaemotional, and epistemic sophistication may contribute to the development of critical thinking in specific domains of knowledge.

It is clear, then, that we assume critical thinking as a unit. A professional who argues well in biology, as well as another who solves problems in physics, another who manages to learn metacognitively in mathematics, or a fourth who performs metaemotive processes in history do not become critical thinkers in these fields of knowledge. The processes of cognitive sophistication, in each of the examples presented, are not sufficient to think critically in each of these fields of knowledge. Critical thinking requires the orchestration of these four processes of sophistication.

## Data Availability

No new data were created or analyzed in this study.
